# Clinical study of comprehensive TCM therapy in the treatment of damp and hot stasis erectile dysfunction

**DOI:** 10.1097/MD.0000000000030776

**Published:** 2022-10-28

**Authors:** Junchao Yao, Baojun Ju, Xiao Li, Miaomiao Ma, Luyu Li, Yongtao Zhang

**Affiliations:** a Henan University of Traditional Chinese Medicine, Zhengzhou, China; b Department of Andrology, The First Affiliated Hospital of Henan University of Chinese Medicine, Zhengzhou, China.

**Keywords:** comprehensive therapy of traditional Chinese medicine, Erectile dysfunction, randomized controlled trial, traditional Chinese medicine

## Abstract

The dilemma of male sexual dysfunction has been the focus of the whole society. Erectile dysfunction (ED) is one of the common sexual dysfunctions, and traditional Chinese medicine therapy has shown potential benefits in the treatment of ED. Comprehensive therapy of traditional Chinese medicine (CTTCM) is a comprehensive application of Chinese medicine therapy for auxiliary intervention. Through preliminary clinical observations, we found that CTTCM is simple, safe, and has good efficacy; however, there is a lack of rigorous clinical trials to support its application. Therefore, we designed a randomized controlled trial to evaluate the efficacy and safety of CTTCM for patients with ED of damp-heat stasis type, and to provide evidence for the clinical application of CTTCM and the construction of a traditional Chinese medicine andrology rehabilitation system.

**Methods:**

In this randomized controlled study, 108 eligible patients were assigned 1:1 to the CTTCM group or the tadalafil group. The treatment period was 8 weeks and the follow-up period was 8 weeks. The primary outcome will be the International Erectile Function Score and traditional Chinese medicine Syndrome Score. Secondary outcomes will include the Erection Quality Score, Patient Health Questionnaire-9, and the 7-item Generalized Anxiety Scale. Safety results will include electrocardiogram, blood tests (including blood, liver and kidney function), urine and stool. International Erectile Function Score-5, traditional Chinese medicine Syndrome Score, Erectile Quality Score, Patient Health Questionnaire-9, 7-item Generalized Anxiety Scale and all Safety outcomes will be conducted at baseline, 2th, 4th, 6^th^, and 8th week. Follow-up results will be assessed at 8th week after 8 weeks’ treatment.

**Discussion:**

This study will provide preliminary evidence for the efficacy and safety of traditional Chinese medicine integrated therapy in the treatment of damp-heat stasis ED. In addition, it also provides a basis for the clinical application of Chinese medicine comprehensive therapy and the construction of Chinese medicine andrology rehabilitation system.

**Trial registration:**

Chinese clinical trial registration identifier, ChiCTR2200062016, registered on July 19, 2022.

## 1. Introduction

Erectile dysfunction (ED) refers to “men’s inability to continuously obtain and maintain sufficient penile erection to complete a satisfactory sexual life.”^[1]^ It is a direct threat, but it can cause greater physical and mental pain to the patient and affect the happiness of the family and the stability of the society. In recent years, more and more studies have been done on ED, but its etiology and pathogenesis are still not completely clear.

As we all know, penile erection is a complex psychophysiological process, which is essentially a series of neurovascular activities. When the brain or penis receive local sexual stimulation, nerve impulses are sent from the lower center of the hypothalamus or sacral medulla to the corpus cavernosum, parasympathetic nerve endings and vascular endothelial cells synthesize and release monoxide under the catalysis of nitric oxide (NO) synthase. Nitrogen, nitrogen enters into smooth muscle cells, activates guanylate cyclase, increases cyclic guanosine monophosphate in smooth muscle cells, which activates proteinase K, acts on calcium ion channels, and reduces intracellular calcium ion concentration, smooth muscle cell relaxation. The smooth muscle of the small arteries and vascular sinuses in the corpus cavernosum relaxes, the vascular sinuses in the corpus cavernosum expand, the arterial blood flow increases, and the corpus cavernosum is congested and swelled. The swollen corpus cavernosum compresses the small veins under the albuginea, closing the venous outflow tract, and the contraction of the pelvic floor muscles can also compress the corpus cavernosum, making it further swell and hard to produce an erection. The sympathetic nerves are excited, the smooth muscles of the arterioles and vascular sinuses contract, the pressure in the cavernous body decreases, the veins open, and the penis begins to weaken.^[2]^

To classify the etiology of ED, it can be roughly divided into organic, psychological and mixed, and most patients often have both psychological and organic factors. Psychogenic ED is also known as psychogenic ED. Psychogenic factors are involved in up to 92.8% of the incidence of ED.^[3]^ Studies have shown that some negative emotions such as fear, anxiety, depression, psychological trauma, etc. can significantly damage erectile function and lead to ED. The main influencing factors are mostly from the social and family environment.^[4]^ Traumatic experience and lack of sexual education are the susceptibility factors for psychogenic ED, and interpersonal problems, family or social pressure and major life events are the predisposing factors for psychogenic ED.^[5]^ Domestic research has found that 72.22% of ED patients are caused by disharmony between husband and wife, low self-esteem, lack of sexual knowledge, mental trauma, and stressful psychological factors.^[6]^

According to relevant foreign studies,^[7]^ Vascular ED accounts for 50% to 60% of ED. vascular ED can be divided into arterial ED, venous ED, and cavernous ED. Arterial factors include any disease that may lead to decreased blood flow in the cavernosal artery of the penis, such as atherosclerosis, arterial injury, arterial stenosis, pudendal artery shunt, and abnormal cardiac function.^[8]^ Venous ED includes venous closure dysfunction or venous leakage from various causes.^[9]^ Common causes of venous lesions include congenital venous agenesis, impaired valve function due to various causes, abnormal venous communicating branches and abnormal shunts caused by surgical treatment of priapism.^[10]^ The insufficiency of penile arterial blood supply is not only caused by stenotic lesions, but may also be caused by changes in blood rheology caused by arterial spasm or endothelial dysfunction.^[11]^ Inadequate penile arterial blood supply is associated with many risk factors, including atherosclerotic hypertension, hyperlipidemia, smoking, diabetes, and pelvic radiation therapy. Studies have shown that diabetes, hypertension, dyslipidemia, obesity and smoking are common risk factors for coronary artery disease and ED.^[12]^ Angiographic studies^[13]^ have shown that many ED patients have varying degrees of stenotic lesions of the arterial system, which ultimately lead to insufficient blood supply to the penis.

Currently, PDE5 inhibitors are the first-line drugs for the treatment of various types of ED, including vascular ED. Studies have shown that its total effective rate for ED is about 80%,^[14]^ but no studies have proved that PDE5 inhibitors can fundamentally improve vascular, nerve and other injuries, so there are still some patients who are more responsive to PDE5 inhibitors. Difference. The effective rate of vacuum suction vacuum constriction device (VCD) in the treatment of ED is about 67%,^[15]^ but the appearance and feel of VCD-induced penile erection are different from those induced by natural erection, and it is not effective for bleeding disorders. VCD may cause discomfort such as petechiae, ecchymosis, and hematoma in patients and those on anticoagulant therapy.^[16]^ Extracorporeal shock wave (ESW) is a special sound wave that carries energy.^[17]^ Studies have shown that low-intensity ESW combined with drugs can effectively improve the local vascular endothelial function and nerve function of the penis, which is beneficial to increase the vascular congestion in the cavernous body of the penis. The amount and then increase the hardness of penile erection.^[18,19]^ However, the long-term efficacy, safety, and adverse reactions of ESW in the treatment of ED still require long-term follow-up, and large-scale clinical trials have not been carried out in China. Minimally invasive interventional therapy is a treatment method for arterial ED.^[20]^ Some small-scale clinical studies abroad have shown^[21,22]^ that interventional therapy can significantly improve erectile function in patients with arterial ED. However, interventional therapy cannot solve arterial ED with microvascular disease. At the same time, this treatment method has problems such as postoperative vascular restenosis and reduced long-term efficacy. Its efficacy and possible adverse reactions are still unclear. Penile vascular reconstruction surgery is a microvascular bypass surgery for the treatment of arterial ED, but ED patients with systemic arterial disease or atherosclerosis combined with diabetes, hypertension, dyslipidemia, smoking, aging, etc. Suitable for penile artery bypass surgery.^[23]^ Penile prosthesis implantation has the advantage of not interfering with urination, orgasm, or pleasure after surgery,^[24]^ but this method has the risk of infection and is expensive, so it is not accepted by the Chinese people.^[25]^

About 30% to 40% of ED patients seek more alternative treatments, such as Traditional Chinese Medicine (TCM), due to the inefficiency, invasiveness, high cost or associated side effects and high risks of the above treatments. Chinese medicine treatment of ED has hundreds of years of practical experience. As for the etiology and pathogenesis of ED, physicians in the past dynasties believed that it was closely related to the deficiency of the kidney. This is the basis for guiding clinical medication.^[26]^ According to a study of 1564 cross-sectional study samples and 481 case-control study samples in China,^[27–30]^ damp-heat, phlegm-dampness, and blood stasis accounted for more than half of the total number of ED patients. At the same time, studies^[31]^ have shown that the blood of ED patients is highly viscous. Modern pharmacological experimental studies^[32,33]^ found that Taohong Siwu Decoction can effectively speed up the blood flow rate of arterioles and venules, expand the microvessels, increase the diameter of the microvessels, increase the circulating blood volume, prolong the thrombosis time and prolong the blood stasis model. It can also significantly reduce the whole blood specific viscosity, plasma specific viscosity and serum specific viscosity of blood stasis model rats, thereby effectively improving blood concentration, viscosity, coagulation and aggregation, and inhibiting platelet aggregation. The mechanism research of modern Chinese medicine in the treatment of ED mainly focuses on the effects on nitric oxide synthase/cyclic guanosine monophosphate pathway, regulation of corpus cavernosum smooth muscle relaxation, antioxidant and organ protection mechanisms.^[34]^ Through the preliminary small sample clinical research, we found that TCM comprehensive therapy is simple, safe and effective in the treatment of ED. However, current clinical studies have limitations in methodology and sample size, and lack sufficient data on TCM integrated therapy as adjuvant therapy versus western medicine for ED. Therefore, we aimed to design a randomized controlled trial (RCT) to evaluate the efficacy and safety of comprehensive therapy of TCM (CTTCM) in ED, and to promote the establishment and improvement of the concept of andrology rehabilitation in TCM.

## 2. Methods and Analysis

### 2.1. Design

**Figure 1.**
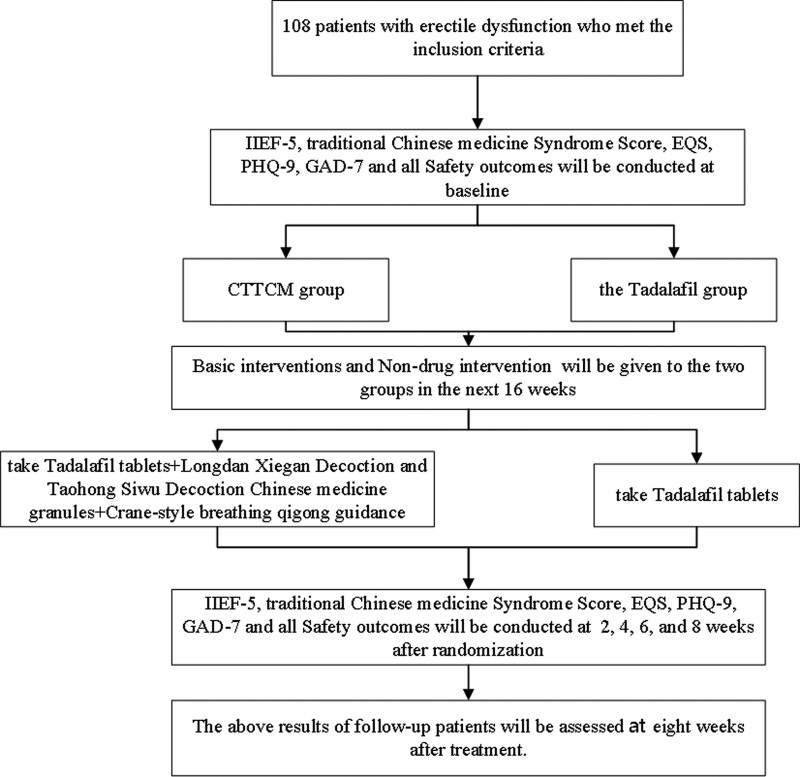
Research flow chart.

An 8-week, single-center, rater-blinded, RCT is currently underway. To evaluate the efficacy and safety of Chinese medicine comprehensive therapy combined with tadalafil tablets and tadalafil tablets in the treatment of ED. The trial will be conducted in the Andrology Department of the First Affiliated Hospital of Henan University of Traditional Chinese Medicine. All participants were required to provide written informed consent prior to entry into the trial. The test flow chart is shown in Figure 1.

### 2.2. Ethical approval

This study protocol was carried out in accordance with the principles of the Declaration of Helsinki^[35]^ and has been approved by the Ethics Review Committee of the First Affiliated Hospital of Henan University of Traditional Chinese Medicine (approval number: 2022HL-070-01) and the whole process was followed up. We registered the study with the Chinese Clinical Trials Registry (registration number ChiCTR2200062016).

### 2.3. Participants

After informed consent was obtained, a total of 108 male patients with ED were included in this study. We will post printed recruitment posters in the hospital or post recruitment information on hospital social media (QQ, WeChat, Weibo, Douyin) to recruit participants. All participants should meet the diagnostic criteria for ED and the TCM syndrome differentiation criteria for damp-heat stasis. If they meet the research criteria, they will be invited to the Andrology Department of the First Affiliated Hospital of Henan University of Traditional Chinese Medicine for research. Eligible participants will be randomly assigned in a 1:1 ratio to the CTTCM group or the tadalafil group. The course of treatment will last 8 weeks. Participants were followed up for erection 8 weeks after treatment.

#### 2.3.1. Diagnostic criteria.

Diagnostic criteria of idiopathic AZS infertility will be based on the “AUA Guidelines for the Diagnosis and Treatment of Erectile Dysfunction”^[1]^ and “The Routine of Andrology,”^[2]^ the diagnostic criteria are as follows: the penis cannot achieve or maintain a sufficient erection state for satisfactory sexual intercourse, and the course of the disease persists More than 3 months; disease grading standard: according to the International Erectile Function Score (IIEF-5) to assess the patient’s condition. IIEF-5 score ≥ 22 indicates that the patient does not suffer from ED, 12 to 21 points are mild ED, 8 to 11 points are moderate ED, and 5 to 7 points are severe ED.

The TCM diagnosis and syndrome differentiation criteria refer to the “Criteria for Diagnosis and Efficacy of Traditional Chinese Medicine Diseases” issued by the State Administration of Traditional Chinese Medicine^[36]^ TCM syndrome differentiation criteria for impotence and the “Guidelines for the Diagnosis and Treatment of Erectile Dysfunction (Trial Version)”^[26]^ Damp-heat stasis in Chinese Medicine The standard of TCM syndrome differentiation of type ED is as follows: main symptoms: ① Yang is not lifted; ② lift is not firm; ③ firm and short; ② pain, dampness and itching in the scrotum; ③ the scrotum is bulging and painful; ④ the mouth is bitter and the throat is dry; ⑤ the urine is hot and cloudy; the pulse is tortuous, thick, swollen, bruised, purple or black; the tongue is yellow and greasy; the pulse is slippery or stringy; with more than one of the main symptoms ①②③, accompanied by more than one of the secondary symptoms ①②③ and more than one of ④⑤⑥ If you see the tongue and pulse at the same time, you can differentiate the symptoms.

#### 2.3.2. Inclusion criteria.

Participants who meet all of the following criteria will be included. The inclusion criteria are as follows: those who meet the diagnostic criteria of western medicine for ED; those who meet the diagnostic criteria of TCM for ED syndrome of damp-heat stasis; males aged 18 ≤ age ≤ 60 years old, meet the same conditions, and have normal libido; and those who have voluntarily signed the informed consent form.

Exclusion criteria are as follows: patients with severe cardiovascular disease, cerebrovascular disease, liver and kidney insufficiency, hematopoietic system disease, neuropathy; combined with urethritis, acute prostatitis, urinary calculi and other diseases; history of pelvic surgery or within 6 months; long-term use of diuretics, antihypertensives, sedatives, antidepressants, hormones and related drugs; unable to cooperate with mental disorders or cognitive dysfunction clinical treatment; those who do not agree to be included or are unwilling to sign the informed consent form; those who have contraindications to the study medication.

### 2.4. Sample size

The sample size was estimated based on the main study results. Our previous preliminary experiments showed that the effective rate (*P*_1_) of the CTTCM group (treatment group) was 80%, and the effective rate (*P*_2_) of the LOS group (control group) was 50%. The following formula was used to estimate the sample size:


$$\begin{array}{l} n=\frac{{\left(Z_{1-\alpha /2}+Z_{1-\beta }\right)}^{2}\left[P_{1}\left(1-P_{1}\right)+P_{2}\left(1-P_{2}\right)\right]}{{\left(P_{1}-P_{2}\right)}^{2}} \end{array}$$

At a significance level of 5% (α = 0.05, two-sided) and a power of 90% (β = 0.1), the final sample size was set at a total of 108 patients (54 in each group), Assume the dropout rate is 10%.

### 2.5. Randomization and blinding

Qualified subjects who provided informed consent will be randomly assigned to the CTTCM group receiving 8-week Chinese medicine combined treatment and oral tadalafil tablets or the tadalafil group receiving oral tadalafil tablets according to the ratio of 1:1. Randomization will be carried out according to the list of random numbers generated by SPSS 25.0 software (International Business Machine Company of Armonk, New York, NY). Independent researchers will prepare for distribution in an opaque envelope, which contains the distribution serial number, and be responsible for hiding the distribution sequence. Due to the specificity of the intervention, it was not possible to blind the participants and personnel involved in the trial. Results evaluators, data managers and statisticians will not know about the treatment task.

## 3. Interventions

### 3.1. Basic interventions

Both groups will implement basic interventions measures, including improving lifestyle to eliminate negative physical and mental factors, such as reducing stress, reducing excess fat, avoiding smoking or drinking, exercising and keeping a comfortable mood.

### 3.2. Drug intervention

#### 3.2.1. CTTCM group.

Longdan Xiegan Decoction and Taohong Siwu Decoction Chinese medicine granules (drug composition: 6 g of gentian, 9 g of skullcap, 15 g of rehmannia, 10 g of Bupleurum, 9 g of gardenia, 12 g of Alisma, 9 g of psyllium, 9 g of Mutong, 15 g of Angelica, 10 g of Chuanxiong, 15 g of Rehmannia glutinosa, 6 g of peach kernel, 6 g of safflower, 10 g of white peony root, and 6 g of licorice). Participants will be required to take Longdan Xiegan Decoction Hetaohong Siwu Decoction Chinese Herbal Granules 2 times a day, 1 tablet at a time, 30 minutes after breakfast and dinner, for 8 weeks. This study will use the granules uniformly formulated by the Chinese Medicine Granules Pharmacy of the First Affiliated Hospital of Henan University of Traditional Chinese Medicine. The above crude drug is extracted into granules, and the production process conforms to the good manufacturing practice standard.

#### 3.2.2. Tadalafil group.

The participants in this group need to take Tadalafil tablets (Shendu), specification: 5 mg/tablet. Dosage: Take one tablet daily, 5 mg orally, orally after dinner. Tadalafil Tablets (Shendu) will be produced by Qilu Pharmaceutical (Hainan) Co., Ltd., with the approval number: Guoyao Zhunzi H20193313. Eight weeks is a course of treatment, and participants will receive a course of drug treatment. All medications that will be used should have the same lot number.

### 3.3. Non-drug intervention

#### 3.3.1. CTTCM group.

Crane-style breathing qigong guidance method (patients lie supine with eyes closed, the whole body is in a calm state, breathe through the nose, with deep inhalation, the lower limbs are in the order from bottom to top, from the toes to the instep and the calf., Thighs gradually contract until the perineum, then lift the anus upwards, hold your breath for 3 to 5 seconds at the end of inhalation, and then begin to exhale, the lower limbs begin to relax, and the anus is relaxed at the same time. The whole process requires the patient to hold their breath, concentrate, and imagine themselves The consciousness moves with the trajectory of the lower limbs. Cycle 15 times before going to bed every night).

#### 3.3.2. CTTCM group and tadalafil group.

*Exercise*: jogging or brisk walking can be used, the speed is about 8 km/h, 5 times per week, and the total time per week is about 150 min.

*Dietary guidance*: The diet should be light, avoid frying, greasy and spicy hot and dry products, and take in sufficient nutrients such as vitamin E, water and zinc. The appropriate intake of total water is 3.0 L/d, which can be added or subtracted according to individual differences and exercise. The recommended dietary intake (RNI) for zinc is 15 mg/d, and zinc-rich foods such as lean beef, turkey, seafood, cereals, and beans are recommended.

*Psychological counseling*: Instruct patients and their spouses to practice behavioral therapy such as sexy concentration training. When couples come to see the doctor together, they should give appropriate education to the woman, instruct the woman to be considerate, and understand men through empathy, so that the patient can gain understanding and warmth. At the same time increase patient’s self-confidence and courage.

### 3.4. Provisions for combined treatment

During the research period, it is forbidden to add other Chinese and western medicines or intervention (such as acupuncture, cupping, massage, etc.) which may interfere with the experiment. For participants suffering from other preexisting diseases, if the combined treatment cann’t be interrupted, the name, dose and frequency of the combined interventions should be recorded in detail. If disease worsens during the study period, participants can withdraw from the study and use other treatments. This case will be regarded as an excluded case, and the patient is required to complete relevant examinations and evaluations as much as possible.

## 4. Outcome measures

### 4.1. Primary outcome

The primary outcomes of this trial were the IIEF-5 and TCM syndrome scores at baseline, at 2, 4, 6, and 8 weeks after randomization, and at follow-up 8 weeks after the end of treatment.

### 4.2. Secondary outcomes

Secondary outcomes will include: Erection Quality Score; Patient Health Questionnaire-9; and 7-item Generalized Anxiety Scale. Testing will be performed at baseline, 2th week, 4th week, 6th week, 8th week and eight weeks after randomization. The above results of follow-up patients will be assessed at 8 weeks after 8 weeks’ treatment.

### 4.3. Safety outcomes

Safety results included electrocardiogram, blood tests (including routine blood, liver function, and renal function), urine routine, and stool routine. All safety outcomes will be assessed at baseline, Week 2, Week 4, Week 6, and Week 8. CTTCM is a comprehensive therapy that has been used clinically for many years with a satisfactory safety profile. A general physical examination is performed at each visit. If an adverse event occurs, the clinical investigator will record it in detail on the case report form (including symptoms, time of onset, duration, examination, and results). Serious adverse reactions will be reported to the Ethics Committee of the First Affiliated Hospital of Henan University of Traditional Chinese Medicine and rescue procedures will be initiated immediately.

### 4.4. Quality control, and trial monitoring

Before the start of the experiment, all researchers will receive special training to ensure the research quality. Training includes how to select and exclude participants, how to complete randomization, how to implement interventions correctly, how to record case report forms in a standard way, and how to evaluate results and manage data. Clinical researcher in charge of diagnosis and treatment will be registered Chinese medicine practitioners. In order to improve the compliance of subjects, the researchers will carry out health education and fully respect the right to informed consent of subjects. The original data will be recorded in the Case Record Form, and two data administrators will input the data into the spreadsheet and review the data separately. In order to ensure the objectivity of the data, the evaluation and statistics during the experiment were blind. The chief researcher will supervise the whole trial.

### 4.5. Statistical analysis

The software of Product and Service Solution (SPSS 25.0; International Business Machine Company, Armonk, NY) will make statistical analysis on the data by professionals who have no knowledge of the whole experimental process. And the efficacy and safety analyses will be based on the principle of intention-to-treat for all random participants. Missing values will be processed by multiple interpolations. Data will be presented in the form of average and standard deviation, median and range, or figures and percentages, and different data types will be analyzed by appropriate methods. The measurement data before and after treatment in the two groups were expressed as mean ± standard deviation (s$\bar{x}\pm s$) for statistical description, and two-sided test was used, and *P* < .05 indicated a statistically significant difference. If the data before and after treatment obey the normal distribution and the variance is homogeneous, the *t* test is used for comparison; The rank-sum test (Mann–Whitney *U* test) was used for rank data. All the statistical tests will be conducted by bilateral difference tests. If the *P* value is less than .05, the data difference will be regarded as statistically significant.

## 5. Discussion

The increase in the incidence of ED has been reported all over the world. At present, for the treatment of ED, both Chinese and Western medicine have their own advantages, but they all have certain limitations. Western medicine attaches great importance to the local treatment of the disease site and ignores the adjustment of the patient’s physical state. Although Western medicine has a clear target for ED, such as PDE5 inhibitors and other drugs, the treatment effect is more significant, but there are still ED patients who have this problem. The response to drug-like therapy is poor, and about 20% to 30% of patients have unsatisfactory effects.^[14]^ In addition, the long-term efficacy, safety, and adverse reactions of ESW, interventional therapy, vascular surgery, and penile prosthesis implantation are unclear and still require long-term follow-up, and large-scale clinical trials have not yet been conducted in China. Research.^[15–24]^

TCM has been used in clinical practice for the treatment of ED in men for hundreds of years. With the development of modern TCM, TCM comprehensive therapy plays an important role in male diseases. Syndrome differentiation and treatment is the most representative feature of TCM syndrome differentiation and treatment. Modern TCM scientists believe that damp-heat and blood stasis are important causes of ED.^[26–30]^ Therefore, we took syndrome differentiation and treatment as one of the inclusion criteria for participants, namely the type of damp-heat stasis stagnation. According to the “Guidelines for the Diagnosis and Treatment of Erectile Dysfunction with Integrated Traditional Chinese and Western Medicine (Trial Version),”^[26]^ the recommended Chinese herbal formula for ED of damp-heat stasis type is Longdan Xiegan Decoction and Taohong Siwu Decoction. Longdan Xiegan Decoction can effectively remove dampness and heat in the body of patients with ED. According to modern pharmacological experiments,^[32,33]^ Taohong Siwu Decoction can effectively accelerate the blood flow rate of arterioles and venules, expand the microvessels, increase the diameter of the microvessels, increase the circulating blood volume, prolong the thrombosis time and blood coagulation when acting on the blood stasis model. It can also significantly reduce the whole blood specific viscosity, plasma specific viscosity and serum specific viscosity of blood stasis model rats, thereby effectively improving blood concentration, viscosity, coagulation and aggregation, and inhibiting platelet aggregation. Some studies^[31]^ have shown that the blood of ED patients is highly viscous, so Longdan Xiegan Decoction and Taohong Siwu Decoction can help improve the hemodynamics in ED patients and treat ED of damp-heat stasis type.

The Crane Breathing Qigong Guidance Method is derived from the traditional Health Qigong Wuqin Xi “Bird Xi” (Crane Xi). The Records of the Three Kingdoms records that Wu Qin Xi was created by Hua Tuo during the Three Kingdoms period., which imitates the actions of five animals: tiger, deer, bear, ape, and bird (crane).^[37]^ Some studies have found that practicing Wuqinxi can improve the body’s balance ability and proprioceptive ability, delay free radical damage, enhance the activity of peripheral blood superoxide dismutase, increase the level of sex hormones, and promote blood circulation, thereby delaying aging.^[38,39]^ Domestic studies^[40,41]^ show that traditional Health Qigong can effectively improve male erectile function.

Exercise, diet rehabilitation and psychological counseling are essential parts of Chinese medicine comprehensive therapy. Studies have shown that adult men need regular exercise, which can be in the form of jogging or brisk walking, with a speed of about 8 km/h, 5 times per week, and a total duration of about 150 minutes per week.^[42]^ The diet rehabilitation method mainly selects foods with dietary nourishment and therapeutic effects in order to promote the overall recovery of the body. A study abroad^[43]^ found that long-term poor diet and nutrition can affect vascular endothelial function by potentially modulating NO and reactive oxygen species, as well as microbiota mechanisms, resulting in vascular ED. TCM pays attention to the homology of medicine and food.^[44]^ Medicines can supplement the power of food, and food can help medicines to recuperate. Dietary rehabilitation should provide advice and guidance on the patient’s diet.^[45]^ The diet should be light, fried, greasy and spicy and hot products should be avoided, and sufficient nutrients such as vitamin E, water and zinc should be taken in.

Vitamin E is an antioxidant substance that participates in the metabolism of the human body. A foreign study^[46]^ found that vitamin E can enhance the production of NO in mouse tissues. Nitric oxide is related to male erectile function, so men need to supplement enough. amount of vitamin E. According to a study,^[47]^ the appropriate total water intake for adult males in my country is 3.0 L/d, which can be adjusted appropriately according to individual differences and exercise. Zinc can promote the normal development of sexual organs and normal sexual function, and sufficient zinc in the human body can ensure strong sexual desire, healthy and normal sexual function and reproductive ability. The recommended dietary intake (RNI) of zinc for men over 18 years old in my country is 15 mg/d, which is significantly higher than that of other groups. However, the actual intake of adult men in my country may only be 2/3 of the recommended intake, while lean beef and turkey, seafood, cereals and beans are rich in zinc.^[48]^

Psychological counseling is an important part of health education. Psychological counseling mainly includes behavioral therapy, cognitive therapy, and sexual knowledge education. Behavioral therapy is one of the most widely used psychological counseling methods at present.^[49]^ Through stroking and other sexy concentration training methods, it can reduce the psychological barriers of patients, help patients to enhance their self-confidence, and promote the recovery of their erectile function.^[50]^ When couples come to see a doctor together, they should pay attention to the propaganda and education of the woman, and tell the woman to be considerate and understand men through empathy, so that the patient can gain understanding and warmth, and at the same time enhance self-confidence and courage, so as to promote the patient’s spiritual recuperation and recovery from disease.

Andrology is a medical specialty that explores male sexual organ development, function and dysfunction (andrology disease), and is an emerging discipline based on male reproductive health. Li^[51]^ believes that comprehensive therapy has become the core concept of andrology disease, emphasizing mental and psychological factors and humanistic care, daily life is closely related to the rehabilitation of andrology and the prevention of recurrence. Comprehensive therapy includes medical comprehensive intervention and non-medical comprehensive intervention. Non-medical intervention is also called health management of disease rehabilitation, which has been widely used in andrology. Therefore, the use of TCM comprehensive therapy for the treatment and rehabilitation of ED is of great significance.

At present, there is a lack of high-quality research and evidence on the treatment of ED caused by damp-heat stasis with comprehensive traditional Chinese medicine therapy. Therefore, we are conducting a randomized controlled study to evaluate the efficacy and safety of CTTCM in patients with ED due to damp-heat stasis. This study is a parallel RCT of the positive drug, and we chose tadalafil as the positive drug because the current evidence shows that the use of tadalafil in the treatment of ED can significantly improve erection without serious adverse reactions.^[14]^ We used the IIEF-5 and traditional Chinese medicine Syndrome Score as the primary efficacy indicators in this trial. Erection Quality Score, Depression Self-Assessment Scale, and Generalized Anxiety Scale with 7 symptom items were secondary efficacy indicators, providing more reference for efficacy evaluation. In addition, electrocardiogram, blood tests (including blood routine, liver function, and kidney function), urine routine, and stool routine are all safety indicators. The treatment time was 8 weeks, including 8-week follow-up after treatment, which provided reliable results for prognostic evaluation of efficacy and observation of adverse reactions.

A limitation of this experiment is that the blind method is impossible due to the nature of the intervention. Every effort will be made to ensure that outcome evaluators, data managers and data analysts know nothing about treatment distribution. The inclusion and exclusion criteria were strictly followed to improve the homogeneity of subjects. We hope that the results can provide preliminary evidence for the efficacy and safety of CTTCM in the treatment of ED caused by damp-heat stasis.

## Acknowledgements

We want to thank all the patients and staff who will participate in the trial for their support.

## Author contributions

**Conception:** Junchao Yao, Baojun Ju, Xiao Li.

**Investigation:** Yongtao Zhang.

**Producers:** Miaomiao Ma, Luyu Li.

**Writing – original:** Junchao Yao, Baojun Ju.

**Writing reviews and editors:** Junchao Yao, Xiao Li.
